# A survey of bloodborne occupational exposure protection behavior among qualified Chinese midwives: A cross-sectional study

**DOI:** 10.1016/j.heliyon.2023.e21288

**Published:** 2023-11-08

**Authors:** Yanhua Zhang, Haixia Zhang, Li Li, Jing Li

**Affiliations:** a Nursing Department, Beijing Ditan Hospital Capital Medical University, Beijing, China; b Obstetrics, Beijing Ditan Hospital Capital Medical University, Beijing, China

**Keywords:** Midwife, Blood borne occupational exposure, Knowledge of protection, Protective behavior, Standard prophylaxis

## Abstract

**Background:**

Bloodborne occupational exposure is a major public health concern of the China Health Commission, especially among midwives who are at high risk among healthcare workers. Knowledge of occupational exposure and appropriate protective behaviors play important roles in reducing occupational exposure. The purpose of this study was to understand the knowledge and level of protection against bloodborne occupational exposure among midwives in China.

**Methods:**

This was a multi-center, cross-sectional study. Midwives from hospitals that are members of Infectious Disease Nursing Committee of Chinese Nursing Association were selected as survey participants from February 2019 to February 2022 using a judged sampling method. Data were collected by using a self-developed questionnaire for Chinese midwives to report their current knowledge and behavior related to bloodborne occupational exposure protection.

**Results:**

A total of 2850 questionnaires were distributed and 2742 valid questionnaires were obtained, resulting in an effective rate of 96.21 %. Midwives scored 2742 (6.495 ± 1.529) points for their knowledge about bloodborne occupational exposure protection, with the level and type of hospital being independent factors affecting the midwives' knowledge of bloodborne occupational exposure protection (χ2 = 27.284, P = 0.038; χ2 = 28.374, P = 0.000). Of the midwives, 1460 were qualified for bloodborne occupational exposure protection behavior, with a qualified rate of 53.25 %. Working years (χ2 = 9.372, P = 0.002) and working hours (χ2 = 13.933, P = 0.000) were also the independent factors for bloodborne occupational exposure protective behavior in midwives.

**Conclusion:**

Chinese midwives possess relatively good knowledge of bloodborne exposure protection against bloodborne infectious diseases, but their behavioral level is not optimistic. Improvements to both knowledge and behavioral level of bloodborne occupational exposure protection are necessary.

## 1 Introduction

Chinese medical staff face a high risk of bloodborne occupational exposure that may cause serious harm. Bloodborne occupational exposure occurs when medical and health staff, laboratory staff and relevant supervisors are exposed to blood, bodily fluids or laboratory culture media containing Human Immunodeficiency Virus (HIV), Hepatitis B Virus (HBV) and Hepatitis C Virus (HCV). This occurs while they are engaged in the diagnosis, treatment, nursing, prevention, testing and management of disease [1]. According to the World Health Report [2], 2.5 % of HIV/Acquired Immune Deficiency Syndrome (AIDS) and 40 % of HBV and HCV cases among medical staff are the result of occupational exposure. Zhang. et al. [3] reported that Labor and Delivery (L&D) rooms have the highest rates of skin injury amongst all hospital departments. The L&D room is particularly busy, with midwives exposed on a daily basis to the patient's blood, body fluids, secretions, amniotic fluid, and sharp instruments. Midwives are prone to sharp instrument injury and exposure to blood and body fluids when observing labor and during the delivery process, artificial rupture of membranes, perineal suture, and oxytocin injection. The heavy workload and physical fatigue can affect the accuracy and coordination of diagnosis and treatment procedures, while increasing the possibility of occupational exposure [4,5]. A one-year cross-sectional study by Abere G. et al. [6] found the infection rate of healthcare workers after contact with blood and body fluids was as high as 65.3 %. The study also found that 46.85 % of cases were infected with HBV, 13.99 % with HCV, 9.09 % with *Treponema pallidum* and 4.90 % with HIV [7]. There is a need to strengthen the monitoring of and protection against bloodborne occupational exposure. Compared to the steep increases in hepatitis C, AIDS and other bloodborne infectious diseases [8], Liu X. et al. reported the incidence of hepatitis B has remained consistently high [9]. The risk of infection is inevitably increased after bloodborne occupational exposure [10]. The causes of occupational exposure for midwives are contaminated needle injury and exposure to blood and body fluids. Needle injury and knife scratches occur mostly during lateral episiotomy and suture. During delivery there is long-term exposure to maternal blood, amniotic fluid and vaginal discharge, while postpartum treatment involves exposure to a large amount of medical waste. Consequently, the issues of occupational exposure and occupational protection of midwives has attracted significant attention from national management departments. Countermeasures for the occupational safety protection of nurses have been improved in the Regulations on Nurses [11], and the occupational protection of nurses has now been standardized through legislation. In summary, midwives face a high incidence and risk of bloodborne occupational exposure, but this risk is still preventable and manageable. Improving the knowledge level of bloodborne occupational exposure among midwives and promoting positive protective behaviors are crucial in reducing bloodborne occupational exposure. This study investigated the knowledge and behavior level of bloodborne occupational exposure protection among midwives in China, in order to provide reference for research related to occupational protection among midwives, as well as the education and training on occupational safety protection.

## 2 Materials and methods

### 2.1 Study participants and settings

This cross-sectional study involved multiple centers. By using the judgment sampling method, experts from the Specialized Committee of Infectious Disease Nursing, Chinese Nursing Association selected survey units from member institutions from February 2019 to February 2022, in accordance with the study's objectives. A questionnaire survey was conducted in obstetrics and delivery rooms of hospitals from 29 provinces, cities and autonomous regions of China. Inclusion criteria: (i) In-service registered nurse and midwives in hospital; (ii) being informed about the investigation and prepared to volunteer and participate. Exclusion criteria: (i) Long-term sick leave (>6 months), or no longer practicing as midwife; (ii) refresher midwives and internship midwives. The study complied with the requirements of the Declaration of Helsinki.

### 2.2 Investigation tool

The questionnaire's first draft was created by the researchers after consulting relevant literature. Following consultation with the Specialized Committee of Infectious Disease Care of the Chinese Nursing Association, the questionnaire [12] was designed to assess the knowledge and behavior of Chinese midwives in regard to bloodborne occupational exposure. Based on the Delphi method, the questionnaire was reviewed, revised and finalized after consultation with five experts in related fields, including a hospital management expert, an infection prevention and control expert, an infectious pathologist expert, a nursing expert, and a data statistician. The recovery rates for Delphi expert consultation in the first and second rounds of the questionnaire were 89.69 % and 100.00 %, respectively, while the proposed rates of opinions and suggestions were 78.49 % and 24.56 %, respectively. The expert authority coefficient was 0.865 ± 0.081, and the coordination coefficients for the two rounds of expert opinions were 0.223 and 0.298, respectively. Based on a preliminary survey of 200 midwives, the questionnaire consisted of three sections: (i) general information on midwives, such as their professional title, years of experience, and hospital type. The definitions used were as follows. Junior midwife: mainly engaged in grassroots midwifery work; responsible for assisting doctors to complete maternal delivery; postpartum care and other work. Intermediate midwife: service as a primary midwife teacher; solid theoretical and practical knowledge of maternal and child health care; able to independently complete some complex midwifery tasks. Senior midwife: service as an intermediate midwife; comprehensive study and mastery of relevant knowledge and skills of maternal and child health care; able to independently perform midwife technical services; able to serve in the management of maternal and child health care institutions. Secondary hospitals are regional hospitals in China that provide comprehensive medical and health services to multiple communities and undertake certain teaching and scientific research tasks. These hospitals work under poor economic conditions, have inadequate protective equipment, and service mostly rural parturients who lack prenatal care and maternal training. (ii) basic knowledge of bloodborne occupational exposure, which included knowledge about standard prevention measures, the treatment process for bloodborne occupational exposure, the timing of medication for HIV exposure prevention, and other related information. There were a total of eight items, with one point for every correct answer and 0 points for incorrect answers or “do not know” responses, with a possible full score of 8 points; (iii) judgments about protective behaviors against bloodborne occupational exposure: ① Standard prevention was not achieved (Standard prevention: the patient's blood, bodily fluids, secretions and excretions were infectious and needed to be isolated. Regardless of whether there was significant blood contamination, or whether they came into contact with incomplete skin and mucous membranes, preventive measures had to be taken for people exposed to the above substances); ② Having a bloodborne occupational exposure. Individuals who met neither of the above criteria were deemed qualified, while those who met at least one were deemed unqualified. The questionnaire received a CVI of 0.87 and a Cronbach's α value of 0.82.

### 2.3 Data collection methods

Online questionnaires were distributed and returned by the persons in charge of each province, who had been uniformly trained by the Infectious Disease Nursing Professional Committee of the Chinese Nursing Association. The questionnaire was completed by midwives from the L&D room and the obstetrics department. Midwives were asked to carefully read the purpose of the study and the precautions before filling in the questionnaire. If any questions, they could also consult the person in charge by mail or telephone. The online questionnaire set all questions as mandatory, and could only be submitted after all questions were completed. Logic screening was performed, and questionnaires with obvious errors were excluded. A total of 2850 questionnaires were distributed, of which 2742 were deemed valid, resulting in an effective response rate of 96.21 %.

### 2.4 Statistical methods

SPSS 22.0 was used to analyze the data statistically. Qualitative data were described by using frequency and rate, and comparisons between groups were performed by using the chi-square test. Measurement data were expressed as mean ± standard deviation, and means between groups were compared by using *t*-test or one-way variance analysis. Significant factors in single-factor analysis were analyzed by logistic regression for multivariate analysis, where *p* < 0.05 was considered statistically significant.

## 3 Results

### 3.1 Basic information

Among the 2742 midwives investigated in this study, professional titles included 1934 (70.5 %) junior midwives, 718 (26.2 %) intermediate midwives, and 90 (3.3 %) deputy senior and above. Work experience was thus: 849 (31.0 %) ≤ 5 years, 916 (33.4 %) 6–10 years, 643 (23.5 %) 11–20 years, and 334 (12.2 %) > 20 years. As for hospital types, 1976 midwives (72.1 %) were from tertiary hospitals and 766 midwives (27.9 %) were from secondary hospitals. As for working hours, 1432 midwives (52.2 %) were 8 h, 1065 (38.8 %) were 8–10 h, 165 (6.0 %) were 10–12 h, and 80 (2.9 %) were more than 12 h.

### 3.2 Analysis of factors of knowledge about the bloodborne occupational exposure protection among midwives

The score for midwives' collective knowledge of bloodborne occupational exposure protection was 6.495 ± 1.529 points, with an awareness rate of 81.19 %. Single-actor analysis of the midwives' knowledge of bloodborne occupational exposure protection revealed that professional title (F = 5.159), gender (F = 9.685), age (F = 5.754), working years (F = 7.002), hospital level (F = 2.974), and hospital category (F = 3.889) were all statistically significant factors (p < 0.05) **(**Table 1**).** Logistic regression analysis was used to classify the knowledge score of bloodborne occupational exposure protection as the dependent variable. Independent variables found to have statistical significance in single-actor analysis were included in the regression equation. The regression model and the comprehensive judgment model matched well. Multivariate analysis revealed that hospital grade (χ2 = 27.284, P = 0.038) and hospital category (χ2 = 28.374, P < 0.001) were independent factors for midwives’ knowledge of bloodborne occupational exposure protection **(**Table 2**).**

**Table 1:** Single factor analysis of occupational exposure protection knowledge among Chinese midwives.

Items		Numbers（n = 2742）	Ratio（％ ）	Scores （6.495 ± 1.529）	F	P
Professional tile	Junior	1934	70.5	6.381 ± 1.540	5.159	0.000
	Intermediate	718	26.2	6.757 ± 1.468		
	Sub-senior and above	90	3.3	6.855 ± 1.488		
Gender	Male	24	0.9	5.666 ± 1.992	9.685	0.000
	Female	2718	99.1	6.502 ± 1.523		
Age	18–25	496	18.1	6.252 ± 1.581	5.754	0.000
	26–30	970	35.4	6.373 ± 1.544		
	31–40	938	34.2	6.594 ± 1.489		
	41–50	290	10.6	6.975 ± 1.407		
	51–60	48	1.8	6.645 ± 1.406		
Working Years	≤5 years	849	31.0	6.262 ± 1.579	7.002	0.000
	6–10 years	916	33.4	6.459 ± 1.514		
	11–20 years	643	23.5	6.611 ± 1.494		
	>20 years	334	12.2	6.964 ± 1.381		
Hospital level	Tertiary	1976	72.1	6.453 ± 1.561	2.974	0.003
	secondary	766	27.9	6.587 ± 1.426		
Hospital type	General	2382	86.9	6.441 ± 1.539	3.889	0.000
	Specialized	360	13.1	6.855 ± 1.410		
Working hours	8 h	1432	52.2	6.508 ± 1.526	1.362	0.208
	8–10 h	1065	38.8	6.500 ± 1.516		
	10–12 h	165	6.0	6.393 ± 1.568		
	12小hours and above	80	2.9	6.412 ± 1.681

**Table 2:** Multivariate analysis of occupational exposure protection knowledge among Chinese midwives.

Effect	Model Fit Conditions	Chi-square	p
Intercept	2242.849	.000	
Professional title	2255.686	12.838	0.685
Gender	2260.275	17.426	0.026
Age Group	2273.196	30.347	0.550
Working years	2256.118	13.270	0.961
Hospital level	2270.133	27.284	0.038
Hospital type	2271.222	28.374	0.000

### 3.3 Analysis of protective factors of midwives' bloodborne occupational exposure

Among all midwives in this study, 1460 were qualified for bloodborne occupational exposure protection behavior, with a qualified rate of 53.25 % (1460/2742). As shown in Table 3, single-actor analysis of the bloodborne occupational exposure protection behavior of midwives revealed that professional title (χ2 = 46.614), age (χ2 = 53.043), working years (χ2 = 69.819) and working hours (χ2 = 24.266) were statistically significant factors (*p* < 0.05) Logistic regression analysis was used to determine the protective behavior of bloodborne occupational exposure as the dependent variable, with statistically significant independent variables in single-actor analysis being included in the regression equation. This regression model matched well with the comprehensive judgment model. Multivariate analysis revealed that working years (χ2 = 9.372, P = 0.002) and working hours (χ2 = 13.933, P < 0.001) were independent factors for the protective behavior of midwives against bloodborne occupational exposure **(**Table 4**)**.

**Table 3:** Single factor analysis of occupational exposure protection behaviors among Chinese midwives.

Items		Unqualified（n = 1282）	Qualified（n = 1460）	X2 value	P
Professional Title	Junior	824	1110	46.614	0.000
	Intermediate	402	3168		
	Sub-senior and above	56	34		
Gender	Male	10	14	0.252	0.616
	Female	1272	1446		
Age	18–25	177	319	53.043	0.000
	26–30	435	535		
	31–40	468	470		
	41–50	171	119		
	51–60	31	17		
Working years	≤5 years	305	544	69.819	0.000
	6–10 years	443	473		
	11–20 years	336	307		
	>20 years	198	136		
Hospital level	Tertiary	932	1044	0.485	0.785
	secondary	350	416		
Hospital type	General	1121	1261	0.687	0.407
	Specialized	161	199		
Working hours	8 h	608	824	24.266	0.000
	8–10 h	554	511		
	10–12 h	85	80		
	12 h and above	35	45

**Table 4:** Multivariate analysis of occupational exposure protection behaviors among Chinese midwives.

Items	B	Wald Chi-square	P
Professional Title	−0.161	2.561	0.110
Age Group	−0.008	0.009	0.926
Working years	−0.254	9.372	0.002
Working hours	−0.199	13.933	0.000
Constant	1.234	70.660	0.000

## 4 Discussion

Awareness of bloodborne occupational exposure protection knowledge among midwives is at a moderate level, and hospital factors are important influencing factors.

Midwives’ knowledge of bloodborne occupational exposure protection scored 6.495 ± 1.529 points in this study, with an average awareness rate of 81.19 %. This is in line with an awareness rate of 85.8 % reported by Li [13]. In a study of occupational protection knowledge, Mengjia et al. [14] reported an awareness rate of 75.41 % for AIDS prevention and control knowledge by medical staff in Tianjin. Gurubacharya et al. [15] reported that 61 % of nurses were unaware that hepatitis B and C could spread through needle wounds. A study by Sharma et al. [16] found that only 50.2 % of healthcare workers answered correctly for the spread of disease via needle sticks and sharp instrument injuries. This suggests there is still ample room for midwives to improve their knowledge of bloodborne occupational exposure protection. A study by Karadağ et al. [17] found that midwives who had received operational training in occupational exposure had a significantly lower probability of occupational exposure than untrained midwives. According to the “knowledge, belief and behavior" theory of Qiao et al. [18], good knowledge perception can change behavior. This suggests that medical institutions can reduce the risk of occupational exposure for midwives through phased intensive teaching of knowledge and skills on occupational exposure. We recommended holding diversified and multi-form occupational exposure protection-related knowledge training and assessment, with periodic examinations, in order to expand awareness in this area. The results of this study showed that hospital level and type were independent factors for midwives' knowledge of bloodborne occupational exposure protection. Midwives in maternity and childcare hospitals demonstrated good knowledge of bloodborne occupational exposure protection, perhaps due to their special work environment and duties. Specialized hospitals refer to hospitals specializing in the diagnosis and treatment of a certain disease, and comprehensive hospitals refer to hospitals engaged in the diagnosis and treatment of each disease. Hospitals specializing in maternal and child care are characterized by fine specialty, multiple levels, each with its own emphasis on medical services. Midwives' services for pregnant women and infants are more comprehensive; midwives in such facilities have better mastered bloodborne occupational exposure protection. Studies have also shown that when occupational exposure occurs in specialized hospitals, it is mainly caused by sharp instrument injury, with puncture process (including venipuncture, blood drawing, intramuscular injection, etc.) as the greatest culprit (accounting for 38.3 %). For such hospitals, the implementation of rapid evaluation and increased education of parturients (or family members) before treatment is key to reducing occupational exposure [19]. Hospital administrators should take comprehensive measures to reduce the risk of occupational exposure, such as suitable training of midwives for occupational protection, the strengthening of standard prevention and standardized operating procedures, provision of safety appliances, and improving the monitoring and protection system for occupational exposure to bloodborne pathogens [20]. Midwives in secondary hospitals demonstrate better knowledge of bloodborne occupational exposure protection, perhaps as part of their basic training in this area. Often, if the cervix has already dilated before arrival at the clinic, midwives have insufficient time to properly implement personal protection measures before delivery and may fail to effectively communicate with the patient. Uncooperative parturients may inadvertently cause occupational exposure; for example, sudden pushing away of the midwife during injection accounted for 3.7 % of accidental injuries. A previous study showed that exposure sources in secondary hospitals were bloodborne transmissible hepatitis B (68.06 %), syphilis (6.38 %), and non-infectious sources (12.77 %) [21]. To endure in their professions, midwives in secondary hospitals must master more knowledge of bloodborne occupational exposure and pay greater attention to protecting themselves while serving parturients. Hospital administrators must strengthen the standardized treatment knowledge of occupational exposure of midwives in secondary hospitals. If maternal blood or bodily fluids accidently splash into the eyes, irrigation should be performed immediately according to the conjunctival irrigation method. At the same time, midwives' awareness of self-protection must be enhanced while standard preventive measures are mastered.

### 4.1 The qualified rate of bloodborne occupational exposure protection behavior of midwives is at a moderate level; duration of work experience and working hours are important influencing factors

In this study, 1460 midwives were qualified for bloodborne occupational exposure protection behaviors, with a qualified rate of 53.25 %. Midwives' own occupational protective behaviors influence the occurrence and outcome of occupational exposure. A previous study [22] found that 35 % of midwives were unaware of the standard preventive measures, treatment principles and methods following exposure to risk factors. Only 5 % of midwives consciously took correct protective measures during delivery. Another study [23] found that 50 % of midwives neglected to use protective measures during delivery due to the inconvenience. A study by Kumakech et al. [24] showed that 54 % of midwives were likely to wash their hands, 53.7 % wore gloves, and 44.1 % wore protective clothing when they came into contact with patients. When midwives' occupational exposure protection behavior is unqualified, it increases the incidence and risk of occupational exposure. In March 2009, the Chinese Ministry of Health issued the official “Guidelines for Occupational Exposure Protection against Bloodborne Pathogens” (GBZ/T213-2008) [25]. In order to standardize the management of occupational exposure by medical staff, these guidelines stipulate that follow-up testing should be performed within a certain time period after exposure. Currently, prevention of HIV infection includes routine treatment measures taken within 24 h, as well as post-exposure prophylaxis (PEP) medication. In a study by Phukan [26], 18 % of nurses agreed that PEP would be more effective if taken immediately. In this study, it was found that most midwives had adequate knowledge about PEP and agreed that its effectiveness is greater when taken earlier. This is because delaying PEP can lead to reduced efficacy. The midwife should check the maternal report before operation and perform regular physical examinations and vaccinations. Zeitoun et al. [27] showed that hepatitis B immunoglobulin and antiretroviral therapy were effective at reducing the risk of occupational exposure following occupational exposure to blood. These authors recommended the need for preventive measures against bloodborne occupational exposure.

The results of this study show that working hours and working years are independent factors for the protective behavior of midwives against bloodborne occupational exposure. 8 h/day is a protective factor for midwives to prevent occupational exposure. Lo et al. [28] used logistic regression analysis to show the likelihood of needle stick injury was 1.29-fold higher if working 41–50 h per week and 1.37× times higher if working >50 h per week as compared to normal working hours. Prolonged work can significantly increase the risk of occupational exposure such as sharp instrument injuries. Long working hours are likely to cause reduced memory and attention span [29] and increased fatigue, and hence greater susceptibility to fatigue errors [30]. Hospital administrators are advised to follow the rules of working hours and optimize midwife shift schedules to reduce associated injuries. Midwives with more than 20 years of working experience had better protective behavior against occupational exposure, on account of their rich clinical experience and proper adherence to prevention of occupational protection measures. In agreement with the findings by Ding et al. [31], we found that midwives with fewer years of working experience may have an increased risk of exposure due to their lack of specialist skills and irregular or unreasonable operating procedures. Midwives should wash their hands, wear surgical gowns, and wear sterile gloves before delivering. The midwife stands at the lower right side of the parturient, prepares delivery items, opens the birth bag, inspects whether the items in the bag are complete, and adds necessary items as needed including syringes, anesthetic drugs, neonatal suction tubes, etc. A standard operation procedure for maternal delivery should be developed to standardize and optimize operating procedures, and improve occupational exposure protection to the maximum extent possible. At the same time, midwives must pay attention to the following details of spontaneous delivery to prevent bloodborne occupational exposure in all aspects: (i) The L&D room should be equipped with a disposable delivery kit to reduce the risk of blood impregnation. The midwife must wear a double-layer sterile and protective mask and eye shield during delivery. They must also take additional preventive measures to prevent potential harm to themselves from exposure to maternal blood, bodily fluids and amniotic fluid that may carry viruses. (ii) Minimize the rate of lateral episiotomy, thereby reducing the exposure of newborns to maternal blood infection, as well as reducing wound sutures and needle stick injuries. An anti-stab needle with 1.2 mm tip can be selected during perineal suture repair to provide better tissue penetration, while also minimizing the risk of puncturing the operator's glove and thereby reducing the risk of stab wounds. Midwives should use the device to hold the needle when performing the puncture. (iii) During delivery of placenta or pressing fundus, the midwife should stand beside the parturient and pay attention to reducing blood splashing while maintaining an aseptic environment. Hospital nursing administrators can reduce the risk of bloodborne occupational exposure by improving the medical environment and increasing the allocation of human resources [32]. Midwives should receive continuous training for a full range of occupational protection, thus improving and standardizing their occupational protection behaviors [33].

## 5 Limitations

This study has several limitations. It was conducted to investigate the knowledge and behavior of midwives with regard to bloodborne occupational exposure. However, it did not investigate the attitudes of midwives, and the study categories were limited. Furthermore, male midwives were not analyzed due to their small proportion amongst midwives (<1.0 %). Future research should include additional investigation of the knowledge, attitude and behavior of midwives towards occupational exposure, thus providing evidence-based data for continued reduction of occupational exposure.

## 6 Conclusion

In this study, we investigated the knowledge and protection levels of bloodborne occupational exposure of midwives in midwifery medical institutions in 29 provinces, cities and autonomous regions of China. For this population and discipline, we found that knowledge levels were relatively good, but that behavioral levels were suboptimal. Hospital administrators should strengthen the training of midwives on the protection knowledge of bloodborne occupational exposure, improve the standard prevention during work, and thereby reduce the infection of bloodborne infectious diseases. This study was limited in that it did not explore the establishment of occupational exposure protection system for midwives, the effective use and innovation of protective equipment, nor the development and improvement of protection knowledge system. In addition, it did not address the impact of psychosocial factors on occupational exposure protection for midwives. With the increasing attention to midwifery in China and abroad, related research has gradually increased, primarily focusing on clinical midwives' occupational exposure-related factors and countermeasures. There has been a general move to strengthen occupational protection and to establish and implement an occupational exposure monitoring system, but interventional research on countermeasures is rare. We suggest that researchers conduct a multi-center cross-sectional study examining the impact of midwives’ personality traits, level of psychological flexibility, and social support on occupational protection to achieve optimal outcomes.

## Publisher's note

None.

## Ethics declaratuins

This study complied with the requirements of the Declaration of Helsinki. Human research ethics approval ([2019]No:(076)-1)was obtained from Beijing Ditan Hospital Capital Medical University. Consent was implied through the completion of the survey. The anonymity of participants was assured no identifying information were collected.

## Data availability

Not making data available. The authors do not have permission to share data.

## CRediT authorship contribution statement

**Yanhua Zhang:** Conceptualization, Project administration, Writing – original draft. **Haixia Zhang:** Data curation, Methodology, Resources, Writing – original draft. **Li Li:** Formal analysis, Validation, Writing – original draft. **Jing Li:** Data curation, Project administration, Writing – original draft, Writing – review & editing.

## Declaration of competing interest

The authors declare that they have no known competing financial interests or personal relationships that could have appeared to influence the work reported in this paper.
